# Self-Reported Utilization of International Guidelines for Staging Dogs with Myxomatous Mitral Valve Degeneration: A Survey among Veterinary Practitioners

**DOI:** 10.3390/vetsci10120687

**Published:** 2023-12-02

**Authors:** Marie D. B. van Staveren, Esther Muis, Viktor Szatmári

**Affiliations:** Department of Clinical Sciences, Faculty of Veterinary Medicine, Utrecht University, Yalelaan 108, 3584 CM Utrecht, The Netherlands; m.d.b.vanstaveren@uu.nl (M.D.B.v.S.); e.muis@students.uu.nl (E.M.)

**Keywords:** ACVIM consensus statement, auscultation, cardiomegaly, echocardiography, endocardiosis, murmur, pimobendan, pulmonary edema, questionnaire, standardization

## Abstract

**Simple Summary:**

The American College of Veterinary Internal Medicine (ACVIM) has developed and published international guidelines on common diseases in dogs and cats with the aim of standardizing diagnostic and treatment protocols and facilitating decision-making for veterinary practitioners. Such guidelines first became available for the most common heart disease in dogs, mitral valve leakage, in 2009. An updated version was published in 2019. The present study aimed to answer the question of whether these guidelines are actually used by the intended public more than a decade after their first appearance. An online survey was sent to veterinary practitioners in the Netherlands and Belgium. Of the 524 responses, 363 forms were complete, and only these were used for data analysis. The results showed that 40% of the respondents did not use the international guidelines to stage chronic mitral valve disease. Veterinarians find it difficult to differentiate between those disease stages where echocardiography is needed in dogs that show no clinical signs. In contrast, diagnosing advanced disease, where clinical examination and radiography can be utilized, is said to be easier. We conclude that the number of veterinarians who admittedly do not follow the international guidelines is surprisingly high.

**Abstract:**

Background: ACVIM developed and published guidelines for staging myxomatous mitral valve degeneration in dogs in 2009. An updated version was published in 2019. The present study aimed to investigate whether these guidelines are actually used by the intended public more than a decade after their first publication. Methods: An online survey was distributed among Dutch and Belgian veterinarians through online channels and mailing lists. Results: Of the 524 responses, only 363 complete surveys were analyzed. The ACVIM guidelines are used by 60% of the respondents. Veterinarians find it more difficult to differentiate stage B1 from B2 in asymptomatic dogs compared to diagnosing stage C. Three-quarters of the respondents would recommend echocardiography for an incidentally detected new murmur with an intensity of 3 out of 6 in an adult dog. Two-thirds of the respondents find coughing a convincing finding for stage C disease. Close to half of the respondents associate a horizontal, dull percussion line with pulmonary edema. For confirming cardiogenic pulmonary edema, 98% of the respondents used thoracic radiographs. Conclusions: Veterinary practitioners might not have the expected training and equipment to be able to apply the guidelines in their practices, especially in the differentiation of stage B1 from stage B2.

## 1. Introduction

The American College of Veterinary Internal Medicine (ACVIM) has developed and published guidelines on common diseases for decades [1]. The aim of these consensus statements is to try to standardize diagnostic and treatment protocols and, with this, help practitioners in their daily decision-making. An ACVIM Consensus Statement was published for myxomatous mitral valve degeneration (MMVD), the most common heart disease in dogs, in 2009 [1,2]. Ten years later, in 2019, an updated version was published [3]. According to the ACVIM guidelines, MMVD is staged in four chronologic categories, where A means a predisposed breed without any evidence of the disease. Stage B refers to asymptomatic dogs with a murmur caused by mitral valve regurgitation secondary to MMVD, where B1 dogs have normal-sized hearts and B2 dogs have cardiomegaly secondary to left-sided volume overload, manifested as left atrial and left ventricular dilation [3]. Stage C refers to dogs with present or past clinical signs caused by cardiogenic pulmonary edema, and stage D indicates dogs with therapy-resistant congestive heart failure [1,3].

Our survey-based study aimed to investigate how the ACVIM staging system for MMVD is implemented in veterinary practices in Dutch-speaking Western European countries. 

## 2. Materials and Methods

Veterinary practitioners working in the Netherlands and the Flemish-speaking part of Belgium were asked via various digital channels to participate in an anonymous, web-based survey (Qualtrics, Provo, UT, USA) in Dutch. The questionnaires were sent to individual veterinarians and to veterinary practices through electronic newsletters of the Royal Dutch Veterinary Association (KNMvD), a veterinary event organizing agency (Vitaux), a global veterinary care provider chain (Evidensia), and two pharmaceutical companies (Boehringer Ingelheim and Vetoquinol). In addition, a link to the survey was placed on an online veterinary discussion platform (Het Dierenartsen Gilde, hosted by Facebook). Participation was stimulated by the possibility of winning free entrance to a veterinary cardiology symposium (Hart voor de Praktijk) held in the Netherlands a couple of months later. Questionnaires were collected in the period from 1 July 2021 to 15 August 2021. Participation was voluntary, and an informed consent on the first page of the questionnaire had to be signed in order to proceed with the questionnaire.

The questionnaire consisted of 35 questions in a fixed order, with an obligation to answer each question. Respondents were given the opportunity to revise their completed questionnaire and adjust their answers. The questionnaire consisted of multiple-choice questions with an additional open answer option, short-answer open questions, questions that consisted of a scroll bar that could be set between 0 and 100, indicating a percentage or a number, and Likert scale-based questions, in which the respondents could indicate how much they agreed or disagreed on a 1-to-5 scale: strongly agree, agree, neutral, disagree, and strongly disagree. During manuscript preparation, the categories agree and strongly agree, as well as disagree and strongly disagree, were combined for the majority of questions to increase readability. For variables without a linear distribution, crosstabs were used to explore potential relationships. The Pearson chi-square test was used to determine statistical significance.

Completed surveys were imported from the online platform (Qualtrics, Provo, UT, USA) to a statistical program (Excel, Microsoft v16.78.3, Redmond, WA, USA), where all open answers were grouped in categories. Subsequently, all answers were imported into another statistical program (SPSS Statistics v28, IBM, Chicago, IL, USA) for statistical analysis. Data are presented as percentages. Correlations between various variables were analyzed using the Spearman correlation coefficient (r). The strength of correlation was classified as follows: 0.91–1.00 very strong, 0.71–0.90 strong, 0.51–0.70 moderate, 0.31–0.50 weak, and 0.01–0.30 very weak. The *p*-values less than 0.05 were considered statistically significant. 

## 3. Results

A total of 524 questionnaires were returned. Because of the incompleteness of 161 questionnaires, 363 fully completed questionnaires were analyzed for this study. 

### 3.1. Demography of the Responding Veterinarians, Their Practice Type, and Size

**Figure 1:**
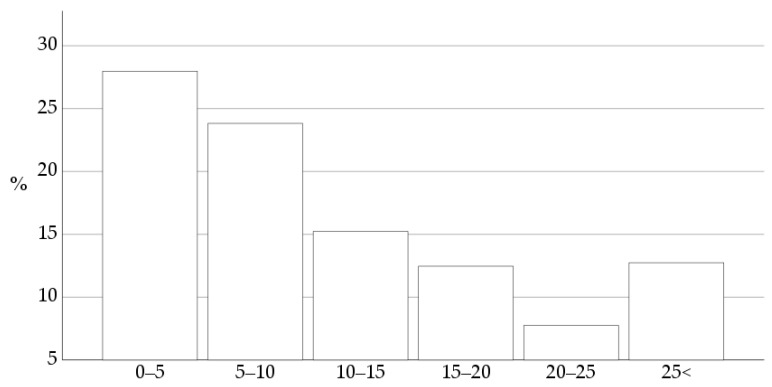
Years of experience working as a veterinary practitioner are shown as percentages of the 363 respondents. The largest group of respondents were those of young graduates with 0–5 years of experience in veterinary practice.

Eighty-five percent of 363 respondents studied veterinary medicine in the Netherlands, 14% in Belgium, and the remaining 1% graduated in Germany, South Africa, or Poland. The number of years in practice is shown in Figure 1.

**Figure 2:**
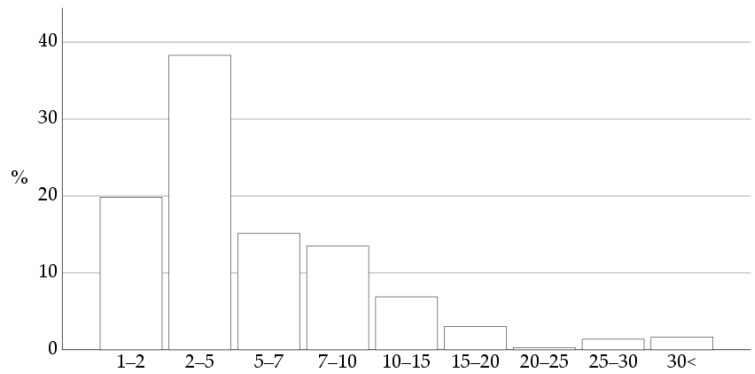
The number of veterinarians employed in the practice where the responding veterinarians work is shown as percentages of the 363 respondents. Practices with 2–5 veterinarians were the most common.

Ninety-seven percent of the respondents work in the Netherlands; the remaining 3% work in Belgium. The respondents’ practice size is shown in Figure 2. 

Eighty-two percent of the respondents work exclusively with companion animals; 9% work in a practice where companion animals contributed to 80–99% of all patients; 4% work in a practice with 40–60% of companion animals; 3% indicated that this percentage was around 60–80%; and only 3% indicated that companion animals were the minority in their practice, between 20–40%.

**Figure 3:**
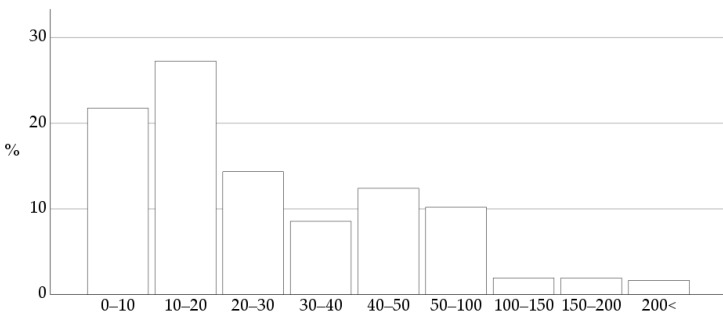
The estimated number of adult dogs in which the 363 respondents diagnose a new heart murmur annually. The most frequent response was 10 to 20 dogs a year.

The estimated number of adult dogs where the respondents detect a new heart murmur annually showed a wide range of variation, as illustrated in Figure 3. 

No association was found between the years of experience as a veterinary practitioner and the estimated number of adult dogs with a new heart murmur annually (*p* = 0.809).

### 3.2. Sources of Continuing Education in Cardiology

Respondents could choose multiple answers as well as add free text. Various sources to stay up-to-date in cardiology were mentioned: 66% learn from peer-to-peer consults, 47% attend refresher courses, 42% let themselves be educated by representatives of the pharmaceutical industry, 37% attend conferences in the Netherlands, 36% read textbooks, 36% call or email a veterinary cardiology specialist, 36% read consensus statements of the ACVIM, and 31% read scientific journal articles. Respondents also mentioned continuing education within their chain (30%), Google (27%), and peer-to-peer coaching (22%). Four percent stated that they do not have time for continuing education in cardiology. 

### 3.3. Staging of Myxomatous Mitral Valve Degeneration (MMVD) According to ACVIM Consensus Guidelines

The ACVIM guidelines for staging MMVD were said to be used by 60% of the respondents. Reasons why the remaining 40% do not use the guidelines were: 53% of them find it difficult to identify the stages, 23% are unaware of such guidelines, and 8% take over the staging from the echocardiography report. The remaining 16% gave various reasons for not following the guidelines, including not seeing the benefit of using them (4%), forgetting to use them (4%), and forgetting what the stages mean (3%). Other answers (5%) included laziness, that it is not in the practice’s protocol to use these guidelines, or that other names or staging systems are used.

No difference was found between the use of the ACVIM guidelines and the number of years in practice, as 66% of recently graduated veterinarians (<5 years) and 58% of experienced practitioners (>15 years) use the ACVIM guidelines (*p* = 0.352).

A significant but very weak positive correlation (r = 0.15, *p* = 0.01) was found between using the ACVIM guidelines and the number of adult dogs the respondents diagnose with a new heart murmur annually.

Respondents who use the ACVIM guidelines read scientific articles more often than those who do not use the guidelines (36% vs. 23%, r = 0.15, *p* = 0.005). Respondents who do not use the guidelines use Google more often (32%) than respondents who do apply the guidelines (24%), but this difference was not statistically significant (r = −0.089, *p* = 0.092).

### 3.4. Clinical Vignette: Diagnostic Recommendations for an Asymptomatic Adult Dog with an Incidentally Found Systolic Murmur with an Intensity of 3 out of 6

The respondents were asked what they would recommend to an owner when they detect a new murmur in an asymptomatic adult dog with a murmur intensity of 3 out of 6. Respondents could choose multiple answers and had the option to fill in free text. Echocardiography was recommended by 77%, thoracic radiography by 47%, blood test for plasma N-terminal pro-brain type natriuretic peptide (NT-proBNP) level measurement by 11%, and 12% would not recommend any diagnostic tests but a recheck when clinical signs appear. The following combinations of diagnostic tests were mentioned: 39% use echocardiography only, 23% use thoracic radiography and echocardiography, 13% only advise thoracic radiography, 3% advise to combine thoracic radiography with NT-proBNP, 3% use thoracic radiography, echocardiography, and NT-proBNP, 2% use echocardiography and NT-proBNP, 2% only use NT-proBNP, and 3% do nothing. Miscellaneous answers such as measuring the respiratory rate at home, starting medication, and a re-check in 6 months were given by 1%. A discrepancy in the answers was found, as 12% of the respondents advised simultaneously a “wait and see” approach and additional diagnostics such as echocardiography or thoracic radiographs.

Respondents who do not use the ACVIM guidelines recommend no further diagnostics, i.e., the “wait and see” approach, significantly more often, compared to the respondents who do use the staging guidelines (25% vs. 10%, r = −0.20, *p* = 0.001). Regarding the choice of diagnostic test, no significant differences were found between respondents who use the ACVIM guidelines and the ones that do not: echocardiography (77% and 77%, *p* = 0.904) or thoracic radiography (49% vs. 43%, *p* = 0.287). The respondents who do not use the ACVIM guidelines recommend plasma NT-proBNP concentration measurement more often than those who use the ACVIM guidelines (17% vs. 7%, r = −0.17, *p* = 0.001).

### 3.5. Differentiating Stage B1 from B2

Of the respondents, 84% recommend differentiating stage B1 from stage B2, whereas 16% do not. When the respondents were asked how they differentiated between these stages, they were allowed to give multiple answers. Echocardiography is used by 73%, thoracic radiography by 46%, and plasma NT-proBNP levels by 3%. The remaining 2% mentioned miscellaneous criteria, such as murmur intensity. The following diagnostic test combinations were mentioned: 37% recommend echocardiography alone, 28% recommend the combination of echocardiography and thoracic radiography, and 17% advise only thoracic radiography. Two percent recommend a combination of either thoracic radiography or echocardiography and plasma NT-proBNP. When using radiography, 95% of the respondents calculate the vertebral heart scale (VHS). Of the 3% who use plasma NT-proBNP, 36% use it in combination with radiography, 27% in combination with echocardiography, 27% with echocardiography and radiography, and 9% use plasma NT-proBNP alone.

The respondents were asked how easy it is for them to diagnose stage B2. To differentiate stage B1 from stage B2, it is reported to be difficult for 43% of the respondents, neutral for 28%, and easy for 29%. The respondents who use the ACVIM guidelines find it easier to determine whether a dog is in stage B1 or B2, compared to those who do not use the guidelines, as evidenced by a weak positive correlation (r = 0.44, *p* < 0.001). Twenty-six percent of respondents who find differentiating stage B1 from stage B2 difficult use thoracic radiography solely or in combination with echocardiography. Interestingly, the ones who find this differentiation easy (61%) or neutral (58%), use thoracic radiography solely or in combination with echocardiography too.

Of the 16% of respondents who stated that they did not differentiate stage B1 from stage B2, 30% find diagnosing stage B2 difficult, 12% respond neutrally, and none of them find it easy. The respondents who do not differentiate stage B1 from stage B2 argued their choice as follows (multiple answers and free text were allowed): 55% indicated the wishes or financial constraints of the owner, 21% thought it would not make a difference for the treatment, 21% indicated they would not do this themselves because they have no ultrasound equipment, 13% indicated insufficient knowledge of the staging criteria, 8% thought it would not be relevant for the prognosis, 8% did not know that a distinction could be made, and 2% indicated limited possibilities.

Fifty percent of those respondents, who do not have time for continuing education in cardiology, do not differentiate between stages B1 and B2. The other 50% use echocardiography less often (19%) to make a difference between stage B1 and B2, compared to the overall percentage of respondents who use echocardiography to make this distinction (73%). Respondents who recommend echocardiography to differentiate stage B1 from B2 use newsletters, attend international congresses, and have subscriptions for online consultations with specialists more often than those who do not recommend echocardiography to differentiate the stages (75% vs. 25%, r = 0.112, *p* = 0.034). No relationship was found between education through peer-to-peer consults and recommending the use of echocardiography (32% vs. 68%, r = −0.016, *p* = 0.244). 

The years of experience as a veterinary practitioner did not influence the ease of determining if a dog is in stage B1 or B2 (r = 0.002, *p* = 0.965).

### 3.6. Recommendations to Owners of Dogs with Stage B Mitral Valve Disease

Respondents could choose multiple answers and add free text for these questions.

#### 3.6.1. Stage B1

Of the respondents, 67% recommend measuring resting or sleeping respiratory rate at home periodically, 61% recommend more often than annual rechecks, 55% recommend making an appointment only when clinical signs appear, 20% recommend nothing additional than the routine yearly health checks, and 7% recommend starting life-long medication. Re-evaluation with echocardiography or thoracic radiography every 6 to 12 months, according to the ACVIM guidelines, is recommended only by 6% and 2% of the respondents, respectively.

#### 3.6.2. Stage B2

Of the respondents, 84% prescribe life-long medication, 75% recommend the owner monitor the resting or sleeping respiratory rate at home, 58% advise a consultation only when clinical signs appear, and 55% recommend more frequent than annual health checks. 

A positive correlation was found between the respondents who use the ACVIM consensus statement and the ones who recommend monitoring respiratory rate at home. Of the respondents who use the ACVIM guidelines for disease staging, 82% recommend monitoring the respiratory rate at home, whereas of the respondents who do not use the guidelines, 65% recommend this (r = 0.19, *p* = 0.001). None of the respondents who use the ACVIM guidelines would wait for the advice of a cardiology specialist before making recommendations to an owner, while 9% of the respondents who do not use the ACVIM guidelines wait for the advice of a specialist before advising an owner (r = −0.24, *p* < 0.001).

Of the respondents who find differentiating stage B1 from stage B2 difficult, 67% recommend that an owner should monitor the respiratory rate at home. In contrast, 88% of the respondents who find diagnosing stage B2 easy would advise monitoring the respiratory rate (r = 0.19, *p* = 0.001).

The number of dogs diagnosed with a new heart murmur annually by the respondent shows a very weak positive correlation with the ease of diagnosing stage B2 disease (r = 0.13, *p* = 0.013). 

### 3.7. Criteria and Test Recommendations for Diagnosing Cardiogenic Pulmonary Edema (Stage C)

Respondents could choose multiple answers for this question. To determine whether a dog is suffering from pulmonary edema, 98% of the respondents use thoracic radiography, 59% use clinical examination, and 20% base the diagnosis on echocardiographic findings. The combination of thoracic radiography and clinical examination was mentioned the most (42%), followed by thoracic radiography alone (34%). The combination of thoracic radiography with echocardiography and clinical examination is used by 13% of the respondents, while 6% use echocardiography combined with thoracic radiography, 1% determine the presence of pulmonary edema solely with clinical examination, and 1% use thoracic ultrasonography. The remaining 3% answered miscellaneous combinations.

Regarding the ease of diagnosing cardiogenic pulmonary edema, 67% of the respondents find it easy, 23% responded with neutral, and 10% find it difficult. A very weak positive correlation is found between the use of the ACVIM guidelines and the ease of diagnosing a dog with stage C disease (r = 0.14, *p* = 0.07). Years of experience do not influence whether respondents find diagnosing a dog in stage C easy or difficult (r = −0.02, *p* = 0.68).

The most convincing findings of pulmonary edema from the medical history were reported to be dyspnea (93%), followed by exercise intolerance (90%), cough (67%), collapse (27%), lack of appetite (21%), and weight loss (19%). 

The most convincing physical examination findings for pulmonary edema in a dog with a heart murmur were found to be increased sleeping respiratory rate at home (98%), dyspnea (93%), increased lung sounds on auscultation (81%), tachycardia (83%), a weak femoral pulse (67%), and tachypnea (57%). A regular pulse is convincing for cardiogenic pulmonary edema for 49% of the respondents, 24% responded with neutral, and 27% find it not indicative for diagnosing stage C disease. 

A horizontal dull line on thoracic percussion and decreased lung sounds on auscultation are convincing findings for pulmonary edema for 46% and 52% of the respondents, respectively. Of the respondents who use the ACVIM guidelines, 69% find a horizontal dull line not fitting with pulmonary edema, while this percentage was 39% of those who do not use the ACVIM guidelines. There is a very slight tendency that respondents who do not use the ACVIM guidelines are more likely to state that a horizontal dull line supports the presence of pulmonary edema compared to the respondents who use the guidelines (r = −0.18, *p* = 0.001). Similar findings were documented for decreased lung sounds on auscultation (r = −0.19, *p* = 0.001). 

A very weak positive correlation was found between the number of dogs diagnosed with a new heart murmur per year and the ease of diagnosing stage C disease (r = 0.14, *p* = 0.006). In addition, a very weak positive correlation was found between the size of the veterinary practice (i.e., the number of veterinarians working there) and the ease of diagnosing stage C disease (r = 0.12, *p* = 0.028).

### 3.8. Wishes of the Respondents to Improve the Management of Their Cardiac Patients

Respondents could choose multiple answers as well as write free text. A quarter of the respondents said that they need nothing else to optimize their management of cardiac cases. More experience in echocardiography is said to be useful by 29% of the respondents, 22% would like more and better refresher courses, 11% wish a shorter waiting time and more direct communication with specialists or better referral options closer to their practice, 10% would like to have a clearer protocol/flowchart about the latest guidelines, 9% wish more motivated owners who are prepared to pay for additional diagnostics, 5% want to improve interpretation skills of thoracic radiographs, and 3% say that they would benefit from more time. Other respondents gave miscellaneous answers, such as having the option to test NT-proBNP in practice or having more knowledge about prognosis.

Respondents who do not use the ACVIM guidelines feel they need more education than those who do (15% vs. 7%, r = 0.13, *p* = 0.012).

## 4. Discussion

According to the authors’ knowledge, this is the first study that evaluates the use of published staging guidelines among the intended users in the field of companion animal cardiology. The aim of the ACVIM consensus guidelines on MMVD was to make the management of dogs with the most common heart disease as uniform and practical as possible, not only for cardiology specialists but also for veterinary practitioners. The first version of the document was published in 2009, 12 years before the conduct of the present survey, and the updated, currently used version was published in 2019 [1,3]. It is striking that 40% of the respondents reported that they do not use the ACVIM guidelines for staging MMVD, and 9% do not know about such guidelines at all. The number of respondents is approximately 12% for the practicing veterinarians who work with companion animals in the Netherlands [4]. The main reported reason for not using the guidelines is having difficulties determining the stages. This suggests that the expertise and equipment required to stage dogs with MMVD might not be readily available in or close enough to an average Western European veterinary practice. 

About 10% of the respondents would not recommend diagnostic testing on a dog with a murmur with an intensity of 3 out of 6, which often represents a moderate to severe mitral valve regurgitation [5,6,7,8,9,10,11,12]. A third of those who do not use the ACVIM guidelines (13% of the total respondents) recommend no diagnostic tests but a “wait-and-see” approach when detecting a new systolic murmur with an intensity of 3 out of 6 in an adult asymptomatic dog. Since the publication of the EPIC trial in 2016, it is known that performing diagnostic imaging tests on these preclinical dogs with a murmur provides not only prognostic information but also a chance for the owner to initiate medical treatment, meaningfully prolonging the subclinical stage and the life expectancy of the pet [5]. Therefore, it is critical to encourage practicing veterinarians to recommend diagnostic imaging tests to owners who have dogs with a likely severe subclinical heart disease, unless the owner expresses financial or other concerns about life-long, twice-a-day medical management. 

There is a marked discrepancy between the frequencies of those saying they do not use the ACVIM guidelines (40%) and those not differentiating between stage B1 and stage B2 (17%). This could mean that the respondents do not know the exact origin of these terms. Echocardiography is the gold standard to establish the cause of a murmur and the disease stage of MMVD. However, in the absence of echocardiography, thoracic radiography in combination with plasma NT-proBNP concentration is recommended along with various variables from the medical history and physical examination, such as murmur intensity [2,3,6,8,9,10,11,12,13]. Because equipment and expertise to perform echocardiography are probably not widely available in first-opinion practices, it is not surprising that 43% of the respondents found differentiating stage B1 from stage B2 difficult. This percentage is only 10% for diagnosing a dog with stage C disease, where physical examination combined with radiography is the gold standard [3]. Unfortunately, there are no data available about the number of practices that own an ultrasound machine or the number of veterinarians who are trained to perform the necessary echocardiographic measurements. Nevertheless, in the countries where the study was performed, echocardiographic evaluation by an EBVS^®^-certified veterinary cardiology specialist is available within a maximum of 2.5 h. 

The ACVIM guidelines advise re-evaluation every 6 to 12 months of dogs in stage B1 by using echocardiography or radiography if echocardiography is unavailable [3]. In our study, only 8% follow this recommendation, but 61% do recommend increasing the frequency of health checks. We can speculate that these respondents would recommend diagnostic imaging only when the intensity of the murmur increases compared to the previous examination, indicating a worsening of the mitral regurgitation [6,8]. Interestingly, two-thirds of the respondents recommend measuring sleeping respiratory rate for owners with stage B1 dogs. This is useful advice, but it focuses on recognizing the onset of clinical signs arising from left-sided congestive heart failure rather than recognizing the more advanced preclinical phase, where so much more profit can be made by timely initiation of medical therapy to delay the onset of cardiac-related clinical signs [3,5]. 

Surprisingly, 16% of the respondents would not recommend medical therapy to a dog in stage B2 MMVD, whereas initiating pimobendan would be indicated [3,5]. In addition, a quarter of the respondents would not educate the owner to count sleeping respiratory rate regularly, whereas tachypnoea in sleep is one of the strongest indicators of the onset of congestive left-sided heart failure [13,14,15,16,17,18]. Moreover, taking a sleeping or resting respiratory rate is easy; it costs only a few minutes of the owner’s time, and it cannot always be replaced by the respiratory rate taken during a veterinary consultation in the practice.

A combination of certain variables from history, physical examination, cardiac biomarkers, and thoracic radiographs can provide moderate accuracy in identifying dogs with stage B2 MMVD [12]. The disease stages can partly be differentiated by the use of plasma NT-proBNP concentration; however, it is subject to individual biological and breed differences as well as comorbidities [19,20,21,22]. Therefore, NT-proBNP values are more reliable when interpreted in combination with other findings, especially thoracic radiographs, and when serial measurements on the same dog are used [12]. Another practical tool in aiding to differentiate dogs in stages B1 and B2 as well as to diagnose stage C disease are breed-specific VHS values [23,24] and the determination of left atrial size on thoracic radiographs [25,26,27,28,29,30].

Over 90% of the respondents are aware of the diagnostic features of cardiogenic pulmonary edema, with dyspnea and increased sleeping respiratory rate being the most frequently mentioned findings. However, much fewer respondents are aware of the indicative value of less specific signs, such as decreased appetite and weight loss [11,31].

Interestingly, two-thirds of the respondents find coughing a convincing clinical sign of cardiogenic pulmonary edema. Though there is still disagreement on this topic among experts, there are several studies that show that cough, particularly if it is chronic, is much more likely to be caused by a primary respiratory disease, such as trachea-bronchomalacia or chronic idiopathic bronchitis, than by pulmonary edema [32,33,34]. Cough receptors are absent in the pulmonary interstitium and alveoli, where pulmonary edema is localized in most cases [33,34].

It is somewhat surprising that a regular heart rate was not found to be a convincing finding of cardiogenic pulmonary edema by many of the respondents. A regular heart rate actually refers to the absence of the irregular heart rhythm, which is caused by respiratory sinus arrhythmia secondary to high vagal tone in healthy dogs and in dogs with preclinical heart disease [12,35,36,37]. The disappearance of respiratory arrhythmia has been shown to be an objective indicator of congestive heart failure secondary to increased sympathicotonus [12,35,36,37]. It is possible that respondents thought of the presence of atrial fibrillation, which typically occurs in large breed dogs with advanced valvular or myocardial disease, or atrial premature complexes, which can be found in small breed dogs with MMVD too [12,38,39].

A rather surprising finding is that almost half of the respondents expect a horizontal dull line on percussion in dogs with pulmonary edema, whereas this is typically caused by the accumulation of free fluid in the pleural space and not by pulmonary edema. This finding can be explained in various ways. One of the explanations is that most veterinary practitioners probably do not perform thoracic percussion on their patients. Another possible explanation is that these respondents mistakenly think that pleural effusion is a consequence of left-sided congestive heart failure in dogs; however, this is only the case in cats [40].

### Limitations

The survey has been distributed online, which might have caused a bias in the number of veterinarians who participated. No questions were asked about the availability of radiography and echocardiography equipment in the practice, the level of expertise in performing and interpreting the imaging findings, if these modalities are unavailable in the practice, and how far the closest location is to perform a basic echocardiographic examination.

The study focused on the usage and perception of the guidelines by veterinary practitioners but did not measure the impact of guideline adherence on patient outcomes. Assessing the effectiveness of the guidelines in improving patient outcomes would provide valuable insights into their real-world clinical relevance and impact.

The study relied on self-reported data from veterinary practitioners, which may introduce bias and inaccuracies in the results. The study did not assess actual knowledge or adherence to the guidelines but rather relied on self-reported utilization. This introduces the potential for respondents to overstate their usage of the guidelines, leading to an overestimation of their actual implementation.

Though the questions were formulated as neutrally as possible, we cannot exclude that the way they were asked influenced the answers. 

Because only 3% of the responses were submitted by veterinarians practicing in Belgium, the results reflect the practice of veterinarians working in the Netherlands. As a consequence, no comparison between the two countries was possible.

## 5. Conclusions

This study suggests that there is a further need to optimize practitioners’ knowledge regarding the application of international standardized guidelines to their patients in everyday practice. Differentiating the preclinical stages seems particularly challenging because of the limited availability of echocardiography. Though diagnosing pulmonary cardiogenic edema seems easier, some misconceptions became apparent, such as about the meaning of cough and thoracic percussion findings. Future considerations can include involving a representative group of practitioners in the development of guidelines and testing the feasibility of the recommendations in first opinion practices before publication of the new edition of the consensus statement.

## Data Availability

The data presented in this study are available on a reasonable request from the corresponding author.

## Abbreviations

ACVIM = American College of Veterinary Internal Medicine; EBVS = European Board of Veterinary Specialization; MMVD = myxomatous mitral valve degeneration; NT-proBNP = N-terminal pro-brain type natriuretic peptide; VHS = vertebral heart scale.
